# Isoflurane inhalation anesthesia should be a new requirement in intracavernosal pressure detection—the gold standard of erectile function assessment

**DOI:** 10.1038/s41598-017-15020-5

**Published:** 2017-11-02

**Authors:** Jinhong Li, Changjing Wu, Fudong Fu, Xuanhe You, Liang Gao, Romel Wazir, Feng Qin, Ping Han, Jiuhong Yuan

**Affiliations:** 1 Andrology Laboratory, West China Hospital, Sichuan University, Chengdu, 610000 China; 2 Department of Urology, Institute of Urology, West China Hospital, Sichuan University, Chengdu, 610041 China; 3 Field Hospital, Zarghun Field, Mari Petroleum Company Limited, Daharki, District Ghotki, Sindh, Pakistan

## Abstract

Intracavernosal pressure (ICP) is gold standard for the detection of erectile function in animals, but no consensus has yet been achieved on what kind of anesthetic protocol should be applied. A total of 16 adult male Sprague-Dawley rats were randomized into two groups. In group A, chloral hydrate was injected intraperitoneally. Rats in group B were induced in 5% isoflurane for 3 min and then maintained in 1.0–1.5% isoflurane. Mean arterial pressure (MAP), respiratory rate (RR) and heart rate were monitored during all experiments. After ICP detection, tail vein and carotid artery blood were collected. The maximum ICP value, MAP and ICP/MAP ratio in group B was significantly higher than in that of group A. The RR in group A was lower than in that of group B, but the heart rate in group A was higher than in group B. There were no significant differences in both pO_2_ and pCO_2_ between groups. While the data showed that animals in group A were relatively hypoxemic. Isoflurane inhalation anesthesia in detection of erectile function could offer a relatively more stable physical state than in that under the effect of chloral hydrate intraperitoneal anesthesia. Isoflurane inhalation anesthesia is more suitable for ICP test.

## Introduction

Penile erection is a complex neuro-vasculo-tissue process and can be influenced by many factors, such as diabetes mellitus and cardiovascular diseases^1^. The detection of erection has close relationship to diagnosis of erectile dysfunction and therapeutic effects of medicine. In human, nocturnal penile tumescence, intracavernous injection test, duplex ultrasound of penis and psychometric tools are available to assess erectile function in male patients^2,3^. International index of erectile function (IIEF) questionnaire, the most popular psychometric test tool, is now widely used to assess patients’ erectile function (EF)^1,4^. But this questionnaire mainly depends on self-description of patients, and thus, could not be applied to animals^5^. Nevertheless, another method can be used to evaluate erectile function of animal—intracavernosal pressure (ICP). ICP detection is more objective and is treated as the gold standard for erectile function in animals^6,7^. Whether disease model is successfully built and whether the treatment is effective mainly depends on ICP. Therefore, the accuracy of ICP is extremely important for animal experiments.

According to reported data, ICP value in normal rats varied greatly among researchers and various anesthetic protocols are widely used^8–11^. At present, no consensus has been achieved as on what kind of anesthetic protocol should be applied to ICP test, and little is known about which protocol is more suitable for ICP detection. Furthermore, whether different anesthetic methods can influence ICP value remains unclear. Thus, in the present study, we investigated the effect of two frequently established anesthesia protocols (inhalational anesthesia and intraperitoneal anesthesia) on ICP detection. The ultimate aim of this study was to offer scientific evidence as for which anesthesia protocol is more reliable for ICP test.

## Results

In order to provide sufficient sedation and analgesia in ICP detection process, additional anesthetics were used when necessary. During the experiments, one rat in group A died of overdose of anesthesia after ICP detection and therefore, no arterial blood analysis data was available for that particular rat.

### ICPmax and ICPmax/MAP

**Figure 1:**
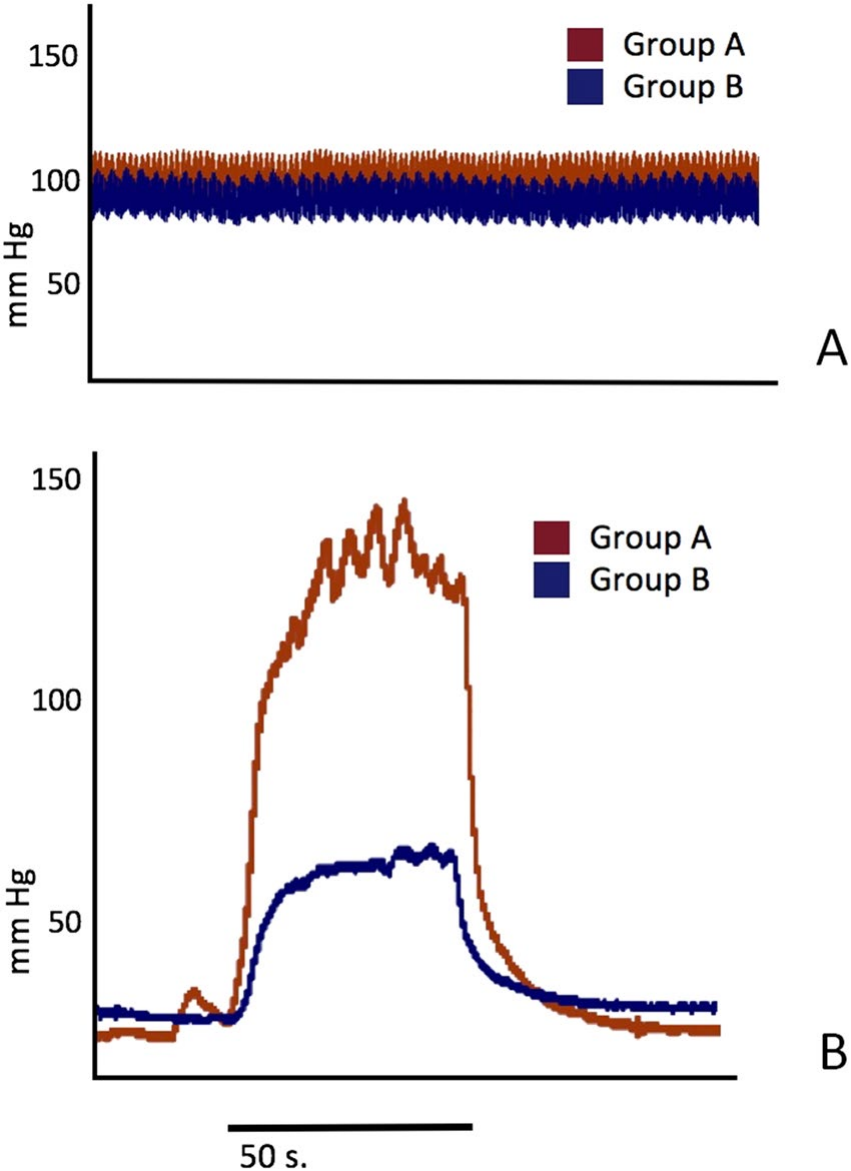
Representative example traces of MAP and ICP.

ICPmax was presented as mean ± S.E.M. The maximum ICP in isoflurane group was significantly higher than in those of chloral hydrate group (109.0 ± 8.9 vs. 54.5 ± 4.5, *p* = 0.0002). The ratio of ICPmax/MAP was presented as median (25% percentage; 75% percentage). After the MAP was adjusted, the erectile function (presented as ICPmax/MAP × 100) were also shown to be significantly higher in group B (60.8 (47.2; 67.0) vs. 111.9 (101.6; 120.9), *p* = 0.0030) (Table 1, Fig. 1).

**Table 1:** ICPmax and ICPmax/MAP.

Group	ICPmax (mmHg)	ICPmax/MAP × 100 (%)
**A** (**N** = **8**)	54.5 ± 4.5	60.8 (47.2; 67.0)
**B** (**N** = **8**)	109.0 ± 8.9*	111.9 (101.6; 120.9)*

ICPmax are presented as mean ± S.E.M. ICPmax/MAP are presented as median (25% percentage; 75% percentage); ICPmax, maximum intracavernosal pressure; MAP, mean arterial pressure.

*p < 0.05 compared with group A.

### Physiological monitoring

During ICP detection, heart rate, respiratory rate and blood pressure were monitored. All data was presented as mean ± S.E.M. The values of respiratory rate and blood pressure in group A were less than those in group B, and both parameters were shown to be statistically significant (53.4 ± 3.0 vs. 60.8 ± 1.7, *p* = 0.0496; 91.6 ± 2.2 vs. 101.6 ± 3.7, *p* = 0.0383; respectively). The group A had a significantly higher heart rate when compared with group B (365.9 ± 23.8 vs. 300.6 ± 12.2, *p* = 0.0341). No significant difference in glucose concentration of rats was found between the two groups (*p* = 0.1111) Table 2.

**Table 2:** Physiological parameters during ICP detection.

Group	Heart rate (/min)	Respiratory rate (/min)	Blood pressure (mmHg)	Glucose (mmol/L)
**A** (**N** = **8**)	365.9 ± 23.8	53.4 ± 3.0	91.6 ± 2.2	5.7 ± 0.4
**B** (**N** = **8**)	300.6 ± 12.2*	60.8 ± 1.7*	101.6 ± 3.7*	5.0 ± 0.3^#^

All data are presented as mean ± S.E.M.

*p < 0.05 compared with group A; ^#^ *p* > 0.05 compared with group A.

### Blood gas analysis

After ICP detection, arterial blood was collected to analyze pO_2_, pCO_2_ and saturated oxygen (StO_2_). Data was presented as median (25% percentage; 75% percentage). Although arterial pO_2_ in group A was lower and pCO_2_ was higher compared with group B, this data did not show significant differences between the two groups (*p* = 0.1602, *p* = 0.1916, respectively). The value of StO_2_ for isoflurane group was determined to be statistically different (observed to be higher) from that of the chloral hydrate group (93.0 (91.6; 94.0) vs. 86.9 (84.0; 88.2), *p* = 0.0084) Table 3.

**Table 3:** Arterial blood gas parameters.

Group	Art. pO_2_ (mmHg)	Art. pCO_2_(mmHg)	StO_2_(%)
**A** (**N** = **7**)	85.4 (79.8; 126.0)	33.8 (32.3; 40.8)	86.9 (84.0; 88.2)
**B** (**N** = **8**)	109.0 (93.7; 133.8)^#^	29.7 (24.5; 36.6)^#^	93.0 (91.6; 94.0)*

All data are presented as median (25% percentage; 75% percentage); Art. pO_2_, arterial pO_2_; Art. pCO_2_, arterial pCO_2_; StO_2_, blood oxygen saturation.

*p < 0.05 compared with group A; ^#^ *p* > 0.05 compared with group A.

## Discussion

ICP is the gold standard for the detection of erectile function in animal experiments, but until now, there is no uniform standard of anesthesia protocols for conducting ICP tests. According to the existing research, the variation of maximum ICP value and the ratio of ICPmax/MAP is huge. Therefore, whether different anesthetic methods have effect on the detection of ICP and which anesthetic protocol is better for testing ICP is still unknown.

In the present study, we evaluated the effect of two widely used anesthesia protocols on physiology and ICP value in rats. Surprisingly, the maximum ICP value in isoflurane anesthesia group was almost two times higher than those in chloral hydrate group. Meanwhile, we noticed that blood pressure in inhalation anesthesia was also higher compared with intraperitoneal anesthesia. Respiratory rate was slower in chloral hydrate group but no significant differences were found in pO_2_ and pCO_2_ between the two groups. Interestingly, isoflurane anesthesia group got a higher StO_2_ than in that of chloral hydrate group. At the same time, we found that the heart rate was faster in chloral hydrate group. This phenomenon might be explained by the reason that respiration seems to be inhibited to a certain extent. Therefore, blood oxygen saturation was significantly reduced and thus heart rate accelerated by feedback mechanism. Additionally, one rat in the chloral hydrate group was found dead because of anesthetic overdose. This may illustrate that inhalation anesthesia could bring about a more stable physiological state than intraperitoneal anesthesia. However, it is still unclear why the relative unstable state caused by intraperitoneal anesthesia has a significant difference in the ICP detection. The effect of respiratory depression, lower blood oxygen saturation and higher heart rate within a short period of time seems to be not enough to result in a significant difference in ICP test. On the other hand, there is another possibility that the difference in ICP detection was caused by erectile nerve blocking brought by the intraperitoneal chloral hydrate.

Although the above discussion does not explain how the two anesthesia protocols affect the ICP detection, we think the finding in present study is of great importance. Our research found that isoflurane anesthesia groups can get higher ICP value, which can provide a much wider range for ICP detection of other experimental groups. ICP test is an optimal method for erectile function, but ICP values reported in previous studies varied widely. Until now, there is no consensus on how much the ICP value should be in healthy rat population. Besides, the expertise and skill of the operating person in ICP detection may also have impact on the data. Therefore, in the detection of erectile function, avoiding the above mentioned factor is needed. Chloral hydrate is a widely used sedative/hypnotic drug in human and has also been applied as anesthetic in animal experimentation^12^. Although intraperitoneal administration of chloral hydrate may result in adynamic ileus or peritonitis, its good anesthetic effect and low price ensure its continued use in researches^13,14^. After ICP detection, all rats need to be sacrificed, and therefore, the application of intraperitoneal injection of chloral hydrate is common in ICP test. However, severe side effects such as death were reported in previous researches and also in present study, so the safety of chloral hydrate may be lower than isoflurane^13,15^.

Although this study is the first one comparing the two anesthesia protocols in ICP detection, there are several limitations. Since isoflurane and chloral hydrate were the most widely used medication for inhalation and intraperitoneal anesthesia in ICP test, other inhalation/intraperitoneal medicines were not considered in our study. In our anesthesia protocols, only one anesthetic dose (chloral hydrate: 3.6%, 1 mL/100 g; isoflurane: 5% for inducing and 1.0–1.5% for maintaining) was used. The effect of different dosage of anesthesia on ICP test is unknown and is still worth discussing. Only normal rats were tested in our study, but rats with impaired erectile function were not included. Considering that the aim of ICP detection in almost all experiments is to distinguish the significance of erectile function among normal, EF-impaired and EF-impaired plus therapy animals, further investigation should be done by comparison of anesthesia protocols on these rats. Because of the small sample size used in our study, the ICP value was only presented as mean +/− S.E.M. Therefore, 5–95 percentile range of ICP value was not considered in the present study. Our preliminary data showed different anesthetic protocols could impact on ICP test, and detail mechanism will be verified by further study in future.

Isoflurane inhalation anesthesia in detection of erectile function could offer a relatively more stable physiological state and higher ICP value than that under chloral hydrate intraperitoneal anesthesia. We conclude that isoflurane inhalation anesthesia is more suitable for ICP test, and also recommend this protocol for future research studies.

## Methods

### Animals

A total of 16 Sprague-Dawley (SD) rats (body weight 280–320 g) were used in this study. All animals were purchased from Dashuo Biological Technology Company, Chengdu, China and housed in Animal Laboratory Center of Sichuan University with an environmentally controlled room at 20–22 degree under a 12 h light/dark cycle. All animals were given food and water ad libitum. All animal experiments complied with the requirements of the Provision and General Recommendation of the National Institute of Health Guidelines for the Care and Use of Laboratory Animals and were carried out with the approval of the Ethics Committee of West China Hospital of Sichuan University.

### Grouping and Anesthesia

Sixteen SD rats were randomly divided into two groups. In group A, chloral hydrate (3.6%, 1 mL/100 g) was injected intraperitoneally and a toe pinch test was used to determine the depth of anesthesia. Each additional 0.3 mL chloral hydrate (3.6%, 1 mL/100 g) was used until satisfactory anesthesia was achieved. Anesthesia was induced by placing rats (group B) in a chamber with 5% isoflurane for 3 min and then maintained in 1.0–1.5% isoflurane through facemask. Air pump (R510–25, RWD life science, San Diego, CA) was used as an air source in the group during anesthesia.

### ICP detection and Monitoring

ICP detection was performed in all rats at the same ambient temperature. After the satisfactory levels of anesthesia induced, rats were fixed on the operation pad. Heart rate/electrocardiogram, respiration and carotid artery pressure were recorded by BL-420F biological function experiment system (Chengdu TME Technology Co., Ltd., Chengdu, China) throughout the whole experiments. The left carotid artery was exposed and cannulated with a 24-G type detaining venipuncture (Closed IV Catheter System, Becton Dickinson Medical Devices Co. Ltd., NJ, USA) filled with 250 IU/ml heparinized saline, and then the catheter was connected to a pressure transducer to measure mean arterial pressure (MAP). The penis was denuded of skin and a 26-G needle (SGJS Medical Equipment Group Co. Ltd., Luohe, China; heparinized with 250 IU/ml heparin) connected to a BL-420F system for recording was inserted into the left side of the penile curs. A midline abdominal incision was made to expose the bladder and prostate. Left cavernous nerve was carefully exposed and isolated. A bipolar platinum electrode (Chengdu TME Technology Co., Ltd., Chengdu, China) attached to an electrical stimulator BL-420F was placed around the nerve for electrical stimulation. The parameters for all rats were 5.0 V, 20.0 Hz, pulse width of 5.0 ms and duration of 50 s^16–18^. Electrocardiogram and respiration were recorded during all the experiments by connecting the BL-420F biological function experiment system. Heart rate and respiratory rate were calculated from the pulsatile pressure wave-form and stretch wave-form. After the ICP detection, tail vein and carotid artery blood were collected for the detection of the blood glucose and blood gas analysis.

### Statistical analysis

All data were first analyzed descriptively and then measures of central tendency and dispersion were computed. All variables were measured on the two-tailed unpaired Student’s *t* test to compare means between groups for normal distribution or Mann-Whitney sign ranked test for abnormal distribution. All data were statistically analyzed using GraphPad prism version 6.0 software (La Jolla; California; USA). A p value of less than 0.05 was considered as statistically significant.
